# Unobstructive Heartbeat Monitoring of Sleeping Infants and Young Children Using Sheet-Type PVDF Sensors

**DOI:** 10.3390/s23229252

**Published:** 2023-11-17

**Authors:** Daisuke Kumaki, Yuko Motoshima, Fujio Higuchi, Katsuhiro Sato, Tomohito Sekine, Shizuo Tokito

**Affiliations:** 1 Research Center for Organic Electronics, Yamagata University, 4-3-16 Jonan, Yonezawa 992-8510, Yamagata, Japantomohito@yz.yamagata-u.ac.jp (T.S.); tokito@yz.yamagata-u.ac.jp (S.T.); 2 Faculty of Education, Art and Science, Yamagata University, 1-4-12 Kojirakawa-machi, Yamagata City 990-8560, Yamagata, Japan; motoshima@e.yamagata-u.ac.jp; 3 Department of Organic Materials Science, Graduate School of Organic Materials Science, Yamagata University, 4-3-16 Jonan, Yonezawa 992-8510, Yamagata, Japan

**Keywords:** sheet-type vital sensor, PVDF, infants and young children, unobstructive vital monitoring

## Abstract

Techniques for noninvasively acquiring the vital information of infants and young children are considered very useful in the fields of healthcare and medical care. An unobstructive measurement method for sleeping infants and young children under the age of 6 years using a sheet-type vital sensor with a polyvinylidene fluoride (PVDF) pressure-sensitive layer is demonstrated. The signal filter conditions to obtain the ballistocardiogram (BCG) and phonocardiogram (PCG) are discussed from the waveform data of infants and young children. The difference in signal processing conditions was caused by the physique of the infants and young children. The peak-to-peak interval (PPI) extracted from the BCG or PCG during sleep showed an extremely high correlation with the R-to-R interval (RRI) extracted from the electrocardiogram (ECG). The vital changes until awakening in infants monitored using a sheet sensor were also investigated. In infants under one year of age that awakened spontaneously, the distinctive vital changes during awakening were observed. Understanding the changes in the heartbeat and respiration signs of infants and young children during sleep is essential for improving the accuracy of abnormality detection by unobstructive sensors.

## 1. Introduction

Unobstructive sensing technology that can non-invasively measure physiological information, such as heartbeat and respiration, is effective in the healthcare and medical fields as a means of measuring vital signs without burdening the subject [1,2,3]. Unobstructive sensing enables long-term and stress-free monitoring; therefore, it is useful from a medical perspective, such as in detecting signs of disease and managing prognoses. These devices are suitable for monitoring elderly people and infants and young children who face difficulty in wearing measuring devices. Long-term monitoring technology is thought to be effective in detecting changes in physical condition and obtaining a deeper understanding of disease condition. Furthermore, by combining long-term monitoring data with AI analysis technology, it will be possible to provide more advanced medical care systems. These techniques are particularly effective in infants and young children who are unable to communicate their symptoms [4,5].

Many unobstructive sensing methods observing vital signs, such as heartbeat and respiration, have been evaluated for integration into unobstructive vital sensors that are based on the detection principle of air pressure [6,7,8], water pressure [9], cameras [10,11], microphones [12], load cells [13,14,15], piezoresistive [16], capacitively-coupled electrodes [17], and piezoelectric films [1,18,19,20,21,22,23,24,25,26,27,28]. Among these, the sheet-type vital sensor using polyvinylidene fluoride (PVDF) as the pressure-sensitive layer is one of the most promising vital sensors from the perspective of sensitivity and adaptability to various measurement environments [1,18,21,22,23,24,25,26,27,28]. When the PVDF pressure-sensitive layer is distorted by an external force, the direction of the dipole inside the PVDF layer changes, and an electric charge is induced in the electrode. This electric charge acts as a sensor-response signal. PVDF is suitable for detecting dynamic external forces because an electric charge is generated by the first derivative of the external force. The response speed is high, on the order of microseconds.

By laying a sheet-type PVDF sensor under a mattress, biological vibration signals, such as heartbeat and respiration, originating from the body of a person sleeping on the bed can be measured. Under normal conditions, the standard heartrate for infants and young children is 100–140 bpm (1.6–2.3 Hz). The heartbeat waveform measured by the sheet-type PVDF sensor is considered to be a composite wave of multiple body vibration components caused by the heartbeat. Therefore, it is necessary to determine the filtering conditions by considering the frequency calculated from the infant’s heartrate and the frequency of the body vibration wave caused by the heartbeat. Similarly, for the respiratory waveform, it is necessary to optimize the filter conditions by considering the frequency calculated from the infant’s breathing rate (20–40 bpm and 0.33–0.66 Hz) and the frequency of the body vibration wave caused by respiration. As the observed frequency bands are different, the heartbeat and respiration signals can be separated by filtering. In particular, the biological vibration signal originating from the heartbeat is called a ballistocardiogram (BCG), which observes the oscillations that occur when blood is rapidly pumped from the heart into the aorta [3].

While there have been many studies conducted on unconstrained vital monitoring in adults, there are only a few relevant reports for infants [29,30,31,32]. A highly sensitive measurement method using a sheet-type PVDF sensor has not been established in unobstructive sensing for infants and young children in previous research. As infants are significantly lighter in weight and have different physiques compared with adults, the optimal measurement conditions and analysis methods are expected to differ from those of adults. A correct understanding of the changes in measurement conditions during the growth stage in infants and young children is essential for improving measurement accuracy.

In this study, an unobstructive heartbeat and respiration measurement system for sleeping infants and young children under the age of 6 years using a sheet-type vital sensor with a PVDF pressure-sensitive layer is demonstrated. VA very-low-noise sheet-type PVDF sensor was achieved by using a differential amplifier circuit. By improving the S/N ratio of the sensor signal, extremely weak heartbeat waveforms on BCGs and phonocardiograms (PCGs) in infants and young children were successfully observed. As BCGs and PCGs have different generation mechanisms, the observed frequency bands are different. Therefore, it is possible to separate them by filtering the sensor signal. It is thought that the observed frequency band for heartbeat waveforms may change depending on the physique of infants and young children. In order to clearly observe the BCG and PCG waveforms, the optimal signal processing conditions of the sheet sensor for infants and young children are discussed based on the measurement data. Heart rate variability (HRV) analysis is possible by extracting the peak interval (PPI) of heartbeat data (BCG and PCG). HRV analysis using the ECG peak interval (RRI) is used in the medical field, so if the PPI can achieve measurement accuracy equivalent to RRI, there is a possibility that sheet-type PVDF sensors can be applied to medical analysis. Electrocardiogram (ECG) and sheet sensor measurements were carried out simultaneously for the subjects. The measurement accuracy of the sheet sensor was verified by comparing the R-to-R interval (RRI) and peak-to-peak interval (PPI) extracted from ECGs, BCGs, and PCGs. The detection of distinctive vital changes during wakefulness can be expected to be applied to sleep analysis in infants. The heartbeat and respiration until awakening in infants were investigated using a sheet sensor. Distinctive vital changes, such as increased respiratory amplitude and heart rate after jerking, were observed with high probability in spontaneously awakened infants under 1 year of age.

## 2. Materials and Methods

**Figure 1:**
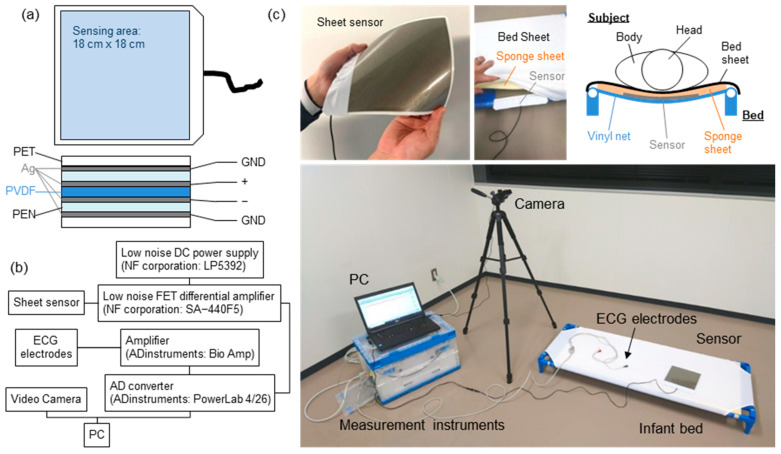
(**a**) Schematic of the device structure of the sheet sensor. (**b**) Experimental setup of the measurement system. (**c**) Photographs of the sheet sensor and measurement system.

The experimental setup is summarized in Figure 1. Figure 1a shows the structure of the sheet-type vital sensors. A polyvinylidene fluoride (PVDF) film (80 µm, KF Piezo, Kureha Corp., Tokyo, Japan) was employed as the pressure-sensitive layer. Ag paste was printed on a polyethylene naphthalate (PEN) film (100 µm) in the shape of an electrode by the screen-printing method, and the sensor was fabricated by laminating the electrodes onto a PVDF film. The outer periphery of the PEN film was covered with a silver electrode film that served as a shielding layer. The sensing area and sensor size were 18 cm × 18 cm and 19 cm × 22 cm, respectively. The thickness of the sensor was less than 1 mm.

Figure 1b shows the configuration of the measurement system. The analog signal of the sheet sensor was amplified using a differential amplification circuit. In this measurement system, 50 times amplification was optimal. However, the amplifier used in this study did not have variable magnification (amplification factor: 100 times fixed). Therefore, an attenuator (6 dB) was connected to adjust the amplification to 50 times. The input impedance of the amplifier was 1 MΩ. The circuit of the sheet sensor did not contain an analog filter circuit, so there was no time lag with respect to ECG data. The captured data were filtered by the software. To obtain a high SN ratio, a differential amplifier circuit and improved shield structure of sheet sensor were employed. Analog signals from the sheet sensor were captured by a PC using an analog-to-digital converter (PowerLab, Bio Research Center Co. Ltd, Nagoya, Japan) with a resolution of 16 bits. All the frequency bands measured from the sheet sensor were recorded without any analog filter circuit. The sampling rate was 1 kHz. The obtained data were analyzed offline using analysis software (LabChart Pro, Ver. 8.1.27), attached to PowerLab.

Figure 1c shows a photograph of the measurement system and the sheet sensor installed on the bed. A cot for infants and young children to take a nap, which is commonly used in Japanese daycare facilities, was used. A sponge sheet with a thickness of 1 cm was laid under the bed sheet, and the sheet sensor was inserted under it. The sheet sensor was placed under the subject’s chest. It was confirmed that the thicker sponge sheet (more than 5 cm) reduced the sensitivity of the sheet sensor. Therefore, in this experiment, a thin 1 cm sponge sheet was employed to minimize the decrease in sensitivity. A similar point was considered regarding the subject’s clothing, but it was not controlled because it was difficult to unify the subject’s clothing. For subjects who gave their consent, ECG data (three electrodes, lead II) were measured simultaneously as a reference device. The situation at the time of the measurement was recorded using a video camera.

Table 1 summarizes the physical information of the subjects and the measurement conditions. Healthy preschool children under the age of six years were measured. ID06 and ID14 were the same subjects, and measurements were performed twice at different times. The participants were asked to come to the laboratory with their parents. The measurements were acquired while they were lying on the bed (shown in Figure 1) installed in the laboratory. In the case of subjects who could not fall asleep, their parents put them to sleep, and after falling asleep, they were transferred to the bed. The measurement was performed in the supine position; however, certain subjects were measured in the lateral position because of the characteristics of the child. Measurements were acquired until the subject woke up spontaneously to observe changes in vital signs until awakening. Younger children tended to demonstrate shorter sleeping times, probably because stable sleep has not yet been established. As the sleep times (measurement times) differed depending on the subject, the measurement times for each subject are listed in Table 1.

## 3. Results and Discussion

**Figure 2:**
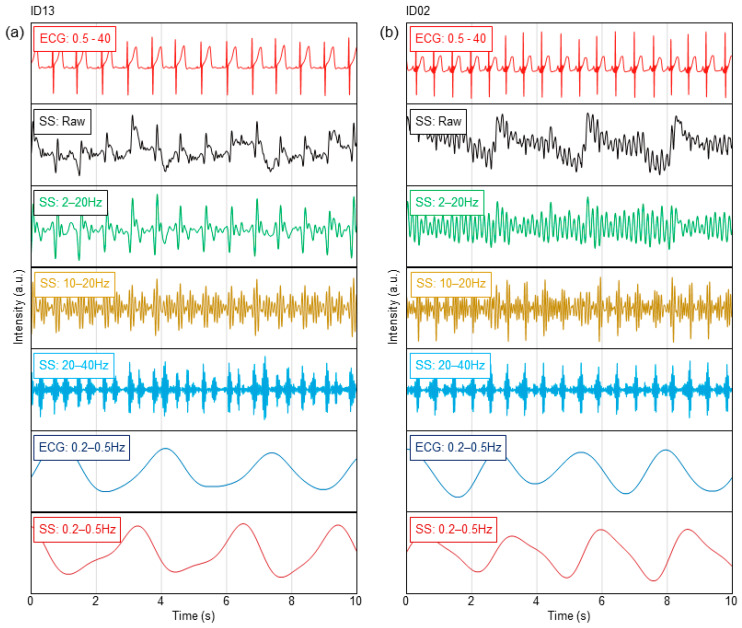
Heartbeat and respiration waveforms in ECG and sheet sensor for (**a**) ID13 and (**b**) ID02.

**Figure 3:**
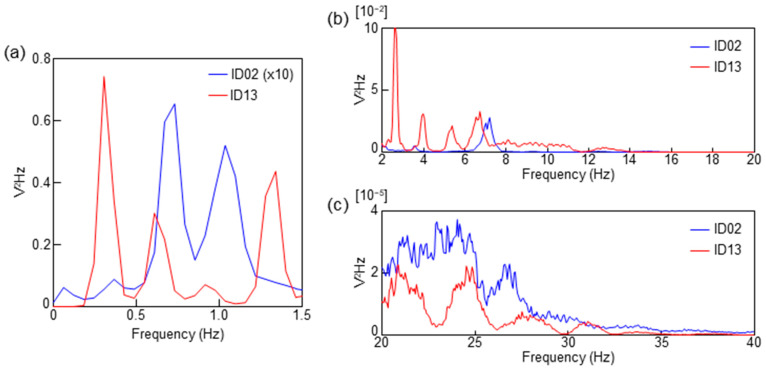
PSD spectra calculated from the sheet sensor data for ID13 and ID02. (**a**) PSD of the frequency range of respiration. The intensity of ID02 was multiplied by 10 in (**a**). (**b**) PSD of the frequency range of BCG (2–20 Hz). (**c**) PSD of the frequency range of PCG (20–40 Hz).

Figure 2 shows the typical results of the vital waveform of a subject whose ECG data and sheet sensors could be measured simultaneously. The typical results of high-age (ID13) and low-age (ID02) subjects are shown in Figure 2a,b. Figure 2 shows the waveform data for 10 s during resting sleep for each subject. The BCG and PCG were filtered in a frequency band well above the heartbeat period, which is sufficient to observe the high heart rates of infants and young children. The ECG was bandpass filtered at 0.5–40 Hz to remove electrical noise. The BCG, PCG, and respiratory waveforms were extracted by bandpass-filtering the raw data of the sheet sensor. The measurement accuracy of the BCG and PCG data was assessed by comparison with ECG data. In adult measurements, the chest-wrapped respiratory inductance plethysmography (RIP) sensors used in polysomnography are generally utilized as reference devices for respiration measurement. However, in this study, it was necessary to completely avoid the risk of respiratory failure due to chest tightening in infants and young children, so these devices could not be used from a safety perspective. As an alternative to the RIP sensor, respiratory waveforms were extracted from ECG as reference data. The ECG waveform includes changes in the impedance of the thorax due to respiratory motion. Therefore, this method is also used in medical equipment to estimate the respiratory waveform. In Figure 3, respiratory waveforms were extracted by filtering the raw ECG data at 0.2–0.5 Hz.

From the results of ID13 in Figure 2a, a clear BCG can be observed by bandpass filtering at 2–20 Hz. The BCG peak is delayed approximately 0.15 s from the R-peak in the ECG in Figure S1a. The BCG waveform was obscured by bandpass filtering at 10–20 Hz, whereas two peaks were observed within one beat. As heart sounds are generally observed in the frequency range above 20 Hz, these peaks are considered to be low-frequency components corresponding to the first sound (S1) and second sound (S2) of the heart [33,34,35,36]. From the waveform filtered at 20–40 Hz, a PCG can be clearly obtained (Figure S1a). In this case, the peak delay of the PCG against the R-peak in the ECG decreased to approximately 0.06 s. The respiratory waveform can be clearly observed by filtering at 0.2–0.5 Hz. Comparing the respiratory waveforms extracted from the ECG, the amplitude periods were similar. From this result, it was confirmed that the respiration waveform could be measured by the sheet sensor. The background noise that increased and decreased simultaneously with the breathing cycle observed in SS at 20–40 Hz in Figure 2a indicated breathing sounds. The timing of the breath sounds coincided with the amplitude phase of the respiratory waveform.

In contrast, a clear BCG was difficult to observe in ID02 when a frequency band below 10 Hz (filtered at 2–20 Hz) was included, as shown in Figure 2b. Distinct BCG peaks were observed by filtering at 10–20 Hz. In this case, the BCG peak was delayed by approximately 0.11 s from the R-peak in the ECG in Figure S1b. The PCG peak was clearly observed in the graph filtered at 20–40 Hz in Figure S1b. Although S2 was weak, S1 and S2 peaks were also observed after filtering at 20–40 Hz. The respiratory waveform was clearly observed by bandpass filtering at 0.2–0.5 Hz, as in the case of ID13. Under filtering conditions at 0.5 Hz or higher, the respiratory waveform was unclear, probably because the separation from the body oscillation component was insufficient.

Figure 3 shows the power spectral density (PSD) computed from the fast Fourier transform (FFT) analysis for 10 min during the sleep of each subject. The raw data of ID02 and ID13 were processed with a 0.05 Hz high-pass filter and subsequently computed with an FFT size of 16K. Figure 3a shows the PSD from 0 to 1.5 Hz, corresponding to the frequency of the respiratory waveform. In Figure 3a, the intensity of ID02 was multiplied by 10 because the respiratory signal of ID02 was weak. Figure 3b,c correspond to the frequency ranges of the BCG and PCG, respectively.

There was a difference in the peak positions of ID02 and ID13, as shown in Figure 3a. ID13 exhibited the highest peak in the frequency band from 0.2 to 0.5 Hz. Furthermore, the PSD peak in the frequency range from 0.2 to 0.5 Hz of ID02 was weak. Two large peaks were observed in the frequency range of 0.5 to 1.2 Hz in ID02. As shown in Figure 2b, a respiratory waveform of ID02 was obtained by bandpass filtering at 0.2–0.5 Hz. The peak in the frequency range of 0.5 to 1.5 Hz was presumably due to a signal caused by body vibration and/or harmonics. The difference in the intensity of respiratory amplitude observed between ID13 and ID02 is thought to be affected by the difference in respiratory momentum due to physique. The amount of respiratory momentum is also affected by the sleep depth. The very low respiratory amplitude of ID02 may indicate that they were in a state of deep sleep [32,37].

In Figure 3b, ID13 exhibits four peaks at approximately 3, 4, 5, and 7 Hz. This is consistent with the results in Figure 2a, which show that the 2–20 Hz filter conditions were suitable for BCG observation in ID13. In contrast, a weak peak at 3.5 Hz and a relatively strong peak at 7 Hz were observed in ID02. This result is consistent with Figure 2b, in which almost no BCG signal was observed in ID02. As the BCG signal of ID02 was clearly observed by filtering at 10–20 Hz, the peak near 7 Hz of ID02 in Figure 3b was presumed to be an unnecessary component caused by body oscillation. The BCG shows the oscillations that occur when blood is rapidly pumped from the heart into the aorta, but the generation mechanism of the BCG waveform has not been elucidated. As thinner tubes tend to reverberate at higher frequencies, it is presumed that the BCG observed at higher frequencies in infants is related to the thickness of blood vessels, the source of the BCG [3,15,38]. Therefore, measuring up to a high-frequency range is necessary when measuring infants and young children using the proposed sheet sensor. In order to clarify this mechanism, further research work for additional verification is essential. A clear difference cannot be observed in Figure 3c in the PCG region.

**Figure 4:**
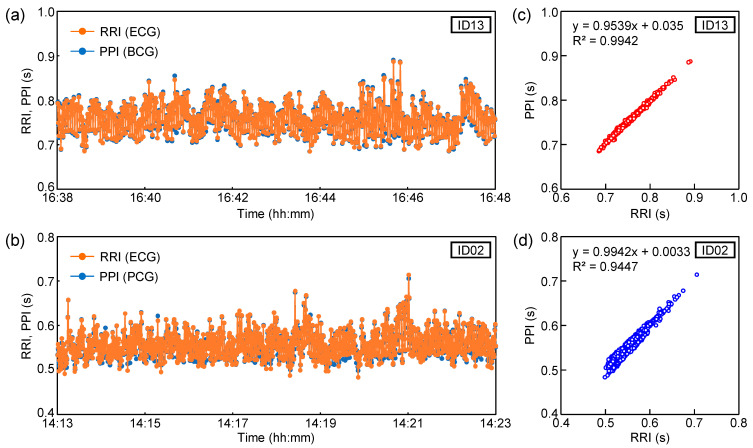
Variations in RRI and PPI over 10 min for (**a**) ID13 and (**b**) ID02. Correlations between RRI and PPI for (**c**) ID13 and (**d**) ID02.

Next, the detection ability of the heartbeat peak interval in the sheet sensor was estimated. Considering the results in Figure 2, the filter conditions with the highest peak detection accuracy in each subject were employed. The heartbeat peak of ID13 was extracted from the BCG filtered at 2–20 Hz. The heartbeat peak of ID02 was extracted from the PCG filtered at 20–40 Hz. As shown in Figure 2b, the BCG signal filtered at 10–20 Hz exhibited poor peak detection accuracy due to the close intensity of the highest and second-highest peaks. In contrast, the PCG signal filtered at 20–40 Hz showed high peak-detection accuracy due to the large difference in intensity between the highest and next-highest peaks. Peak extraction was performed for 10 min during sleep. Peak detection was performed using the cycle calculator function in LabChart software. The peak interval data of the sheet sensor were compared with those of the ECG. The time delay between BCG and ECG in this experimental system was always constant under the same measurement conditions, so no time correction was carried out for ECG and BCG when calculating RRI and PPI. The peak interval of the ECG is shown as the RRI. The peak interval extracted from the sheet sensor (BCG or PCG) is defined as the PPI. Figure 4a,b show the variations in PPI and RRI over 10 min obtained from ID13 and ID02. The horizontal axis in Figure 4a,b indicates the actual time when the data were measured. Figure 4c,d show the calculated correlation coefficient (R) between RRI and PPI for the 10 min data in Figure 4a,b. Only two points in the PPI of ID13 deviated considerably owing to vibration noise from around the bed; thus, those points were excluded from the software. The heart rate peak number extracted in 10 min was 787 points for ID13 and 1075 points for ID02.

The PPI of ID13 remained at approximately 0.7–0.8 s (heart rate: approximately 75–85 bpm). The PPI of ID02 remained at approximately 0.5 to 0.6 s (heart rate: approximately 100–120 bpm). As the subject was in a quiet state of sleep, there was no significant change in PPI during the 10 min period. The RRI and PPI plots almost overlapped for both subjects. From the results in Figure 4c,d, RRI and PPI showed a high correlation coefficient in both subjects. The correlation coefficients, R, of ID03 and ID02 were 0.99 and 0.97, respectively. The high correlation coefficients between RRI and PPI indicate that the heart rate measured by the ECG and sheet sensor almost matched. As shown in Figure 4a,b, the data showed a high correlation coefficient, even for a relatively long 10 min period. It was confirmed that the sheet sensor had high measurement accuracy comparable to ECG [20,35,36]. As our sheet sensor employed a differential amplifier circuit, the elimination of the effects of power-supply noise and the measurement of biological vibrations with a high signal-to-noise ratio are possible. Noise reduction is important for accurate measurements that require high sampling rates, such as PCG.

**Figure 5:**
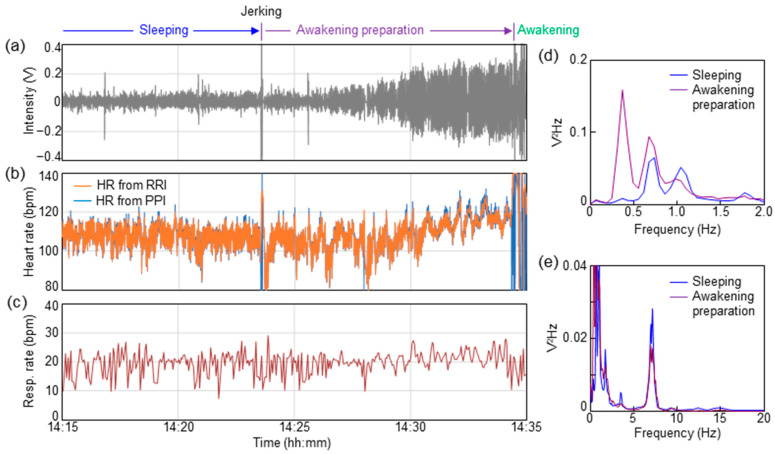
Vital changes during awakening preparation stage for ID02. (**a**) Time dependence of respiratory waveform. (**b**) Time dependence of heart rate calculated from PPI and RRI. (**c**) Time dependence of respiratory rate calculated from BBI. PSD spectra in the ranges of (**d**) respiration and (**e**) BCG.

Finally, the vital changes until awakening in infants using a sheet sensor were investigated. In spontaneously awakened infants under one year of age, the distinctive vital changes during awakening were observed. Similar distinctive vital changes up to awakening were observed in ID02, ID03, ID07, ID10, ID15, and ID17. Figure 5 shows the results of vital changes until awakening for ID02 as a typical result. Figure 5a shows the respiratory waveform for approximately 20 min until awakening. Changes in heart rate were calculated from the PPI and RRI, as shown in Figure 5b. Heart rate was calculated from 60/PPI and 60/RRI, indicating the heart rate per beat. PPI was extracted from PCG, as shown in Figure 4b. Figure 5c shows the change in respiratory rate calculated from the time interval between respiratory peaks (breath-to-breath interval (BBI)). The respiratory rate was calculated from 60/BBI. The horizontal axis in Figure 5a–c indicates the actual time when the data were measured.

As shown in Figure 5a, the respiratory waveform was stable, with an almost constant value, until 14:23:34. A momentary large-body movement (jerking) was observed at 14:23:34. As shown in Figure 5a, the respiratory waveform swung out significantly at this moment. Triggered by this jerking, the amplitude of the respiration signal increased over the next several minutes. Over 10 min of increasing respiratory amplitude, short apnea lasting 5–10 s was observed a few times. As the respiratory amplitude in this apnea was suddenly flattened, it was presumed to be a central apnea, rather than an obstructive apnea. The apnea waveform was not observed during sleep before jerking. An increase in the heart rate was observed with an increase in the respiration amplitude, as shown in Figure 5b. The heart rate before trigger jerking was approximately 110 beats, but it increased to approximately 120 beats immediately before awakening at 14:34:22. The respiration rate also increased by two to three breaths immediately before awakening, as shown in Figure 5c. The subject awakened at 14:34:22.

Figure 5d,e show the results of PSD during 10 min of sleeping (14:13–14:23) and in the awakening preparation stage (14:24–14:34), respectively. The calculations in Figure 5d,e are based on the same procedure as that in Figure 3. The PSD data of sleeping (14:13–14:23) shown in Figure 5d,e are equal to those of ID02 in Figure 3a,b. As a result of comparing the PSD before and after jerking, a shift in the PSD peak can be observed in Figure 5d, indicating the frequency band of respiration. During sleep, PSD peaks were placed at 0.7 and 1.1 Hz. After jerking, the PSD peaks shifted to 0.3 and 0.7 Hz. This change corresponded to an increase in respiration amplitude during the awakening preparation stage, as shown in Figure 5a. The PSD peaks corresponding to the BCG band of 2–20 Hz in Figure 5e showed no significant change before and after jerking.

Similar vital changes in the awakening preparation stage were observed for ID03, ID07, ID10, ID15, and ID17 (Figure S2). Distinctive vital changes in the respiration amplitude, heart rate, and respiration rate after jerking were observed. Interestingly, these subjects were younger than one year of age. Nine subjects under one year of age spontaneously woke up, and six of them showed distinctive vital changes (Figure S2). Although the cause of distinctive vital changes is not understood clearly, a similar tendency was observed in more than 60% of the subjects. As the subjects in forced awakening woke up within tens of seconds, the distinctive vital changes could not be observed. In this study, vital changes before awakening, as shown in Figure 5, were not observed in subjects aged over one year upon spontaneous awakening. This phenomenon may be common in young children under the age of one year.

## 4. Conclusions

Unobstructive heartbeat and respiration measurements of sleeping infants and young children were performed using a sheet-type PVDF sensor. The signal processing conditions and vital changes in the awakening preparation stage from the heartbeat and respiration data of infants and young children were discussed. In infants, the BCG waveform appeared in a higher frequency band, and the filter conditions changed depending on age. PCG was observed under the filter conditions of 20–40 Hz. The difference in the signal processing conditions was caused by the physique of the infants and young children. The PPI extracted from the BCG or PCG during sleep showed a high correlation with the RRI extracted from the ECG. Distinctive vital changes were observed during the preparation stage. An increase in respiration amplitude, heart rate, and respiration rate after jerking was observed. These phenomena were common in children under the age of one year. The detection of distinctive vital changes during wakefulness can be expected to be applied to sleep analysis in infants. The heartbeat waveform and respiratory waveform measured from the sheet sensor contained a lot of information about physical condition. As the human body is in a resting state during sleep, data with less noise and artifacts can be obtained. By understanding the changes in heart rate and breathing patterns during sleep, it is possible to improve the accuracy of detecting abnormal signals. Clinical research involving a large number of subjects is essential to improve the measurement accuracy of unobstructive measurement technologies. Analysis methods to estimate the sleep stage from changes in vital signs have been researched. By combining these analysis methods, unobstructive sensing enables the long-term, stress-free analysis of more comprehensive sleep states in infants and young children.

## Figures and Tables

**Table 1:** Physical information regarding the participating subjects and measurement condition.

Subject No.	Gender	Age/Months	Height (cm)	Weight (kg)	ECG	Awakening	Measurement Duration (min)
ID01	Male	0/7	68	7.6	With	Spontaneously	33
ID02	Male	0/11	-	7.9	With	Spontaneously	29
ID03	Female	0/11	62.2	8.5	Without	Spontaneously	19
ID04	Male	1/8	84	13	With	Forced	31
ID05	Male	0/11	70	9	Without	Forced	64
ID06	Female	2/6	96	12.5	Without	Forced	68
ID07	Female	0/7	-	7	Without	Spontaneously	54
ID08	Female	4/11	105	17.5	Without	Forced	65
ID09	Male	3/3	95	15.7	With	Forced	53
ID10	Male	0/10	75	10	Without	Spontaneously	22
ID11	Female	1/2	72	10.5	With	Forced	35
ID12	Female	1/6	77	9.5	With	Spontaneously	31
ID13	Female	5/8	104	16	With	Forced	56
ID14	Female	3/2	96	14.5	With	Forced	52
ID15	Male	0/11	72.8	9.2	With	Spontaneously	29
ID16	Male	5/2	110	19	Without	Spontaneously	34
ID17	Female	0/5	65	6.7	With	Spontaneously	22
ID18	Female	1/7	-	8.2	With	Forced	58
ID19	Male	3/11	93.7	13	With	Forced	60
ID20	Female	0/8	67	8.9	With	Forced	24
ID21	Male	0/3	57	4.7	With	Spontaneously	7
ID22	Male	1/10	78	9.6	With	Forced	42
ID23	Female	1/8	84.4	10	With	Spontaneously	54
ID24	Male	0/9	71	8.7	With	Spontaneously	28

## Data Availability

The datasets used and/or analyzed during the current study are not publicly available, but are available from the corresponding author on reasonable request.

## Supplementary Materials

The following supporting information can be downloaded at: https://www.mdpi.com/article/10.3390/s23229252/s1. Figure S1: Heartbeat waveforms in ECG and sheet sensor for (a) ID13 and (b) ID02.; Figure S2: Time dependence of respiration waveform, heart rate, and respiratory rate during awakening preparation stage for ID03, ID07, ID10, ID15, and ID17.
