# Supplementary material for: Unobstructive Heartbeat Monitoring of Sleeping Infants and Young Children Using Sheet-Type PVDF Sensors
Source: Sensors (Basel). 2023 Nov 17;23(22):9252. doi: 10.3390/s23229252 (PMC10674719; doi:10.3390/s23229252)
Supplement: Supplementary file 1 [file sensors-23-09252-s001.zip › sensors-2664787-supplementary.pdf]

Supplementary Materials

Unobstructive Heartbeat Monitoring of Sleeping Infants and Young Children Using Sheet-Type PVDF Sensors

Daisuke Kumaki[1*], Yuko Motoshima[2], Fujio Higuchi[1], Katsuhiro Sato[1], Tomohito Sekine[1,3], Shizuo Tokito[1,3]

[1] Research Center for Organic Electronics, Yamagata University, 4-3-16, Jonan, Yonezawa, Yamagata, 992-8510, Japan

[2] Faculty of Education, Art and Science, Yamagata University, 1-4-12 Kojirakawa-machi, Yamagata City, Yamagata 990-8560, Japan

[3] Department of Organic Materials Science, Graduate School of Organic Materials Science, Yamagata University, 4-3-16 Jonan, Yonezawa, Yamagata 992-8510, Japan

[*] **Corresponding Author:** E-mail: d_kumaki@yz.yamagata-u.ac.jp

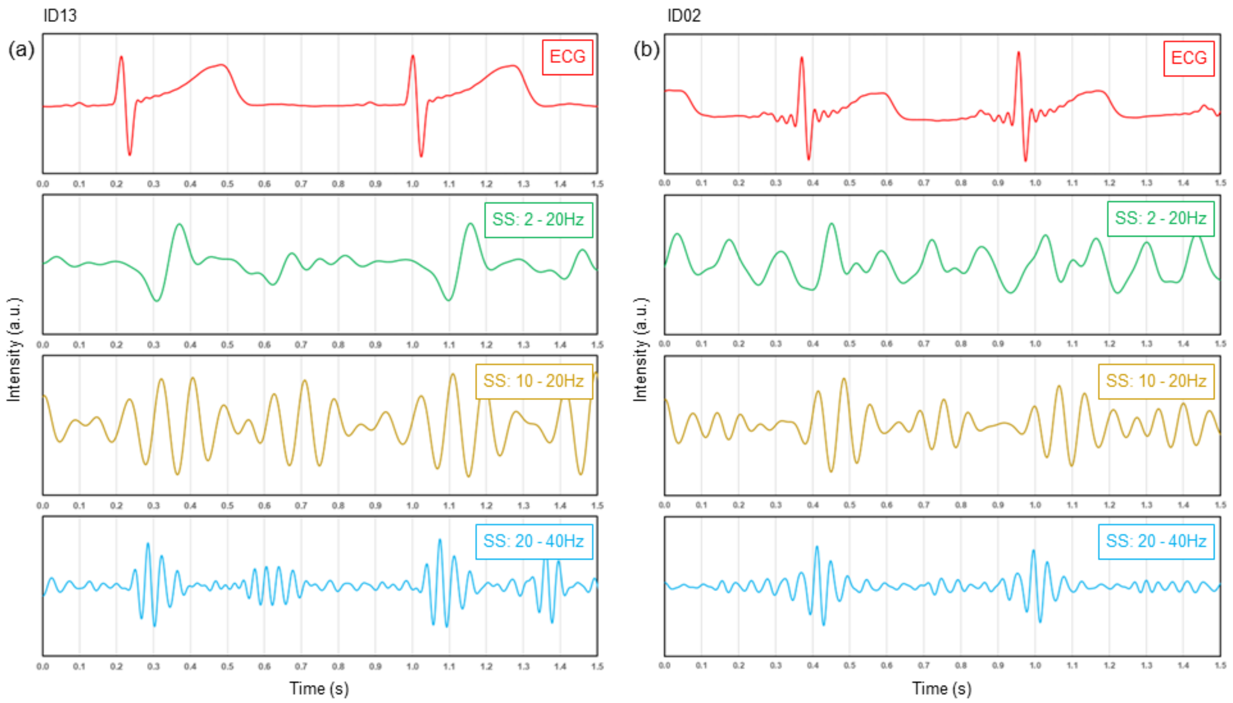

Supplementary Figure S1. Heartbeat waveforms in ECG and sheet sensor for (a) ID13 and (b) ID02.

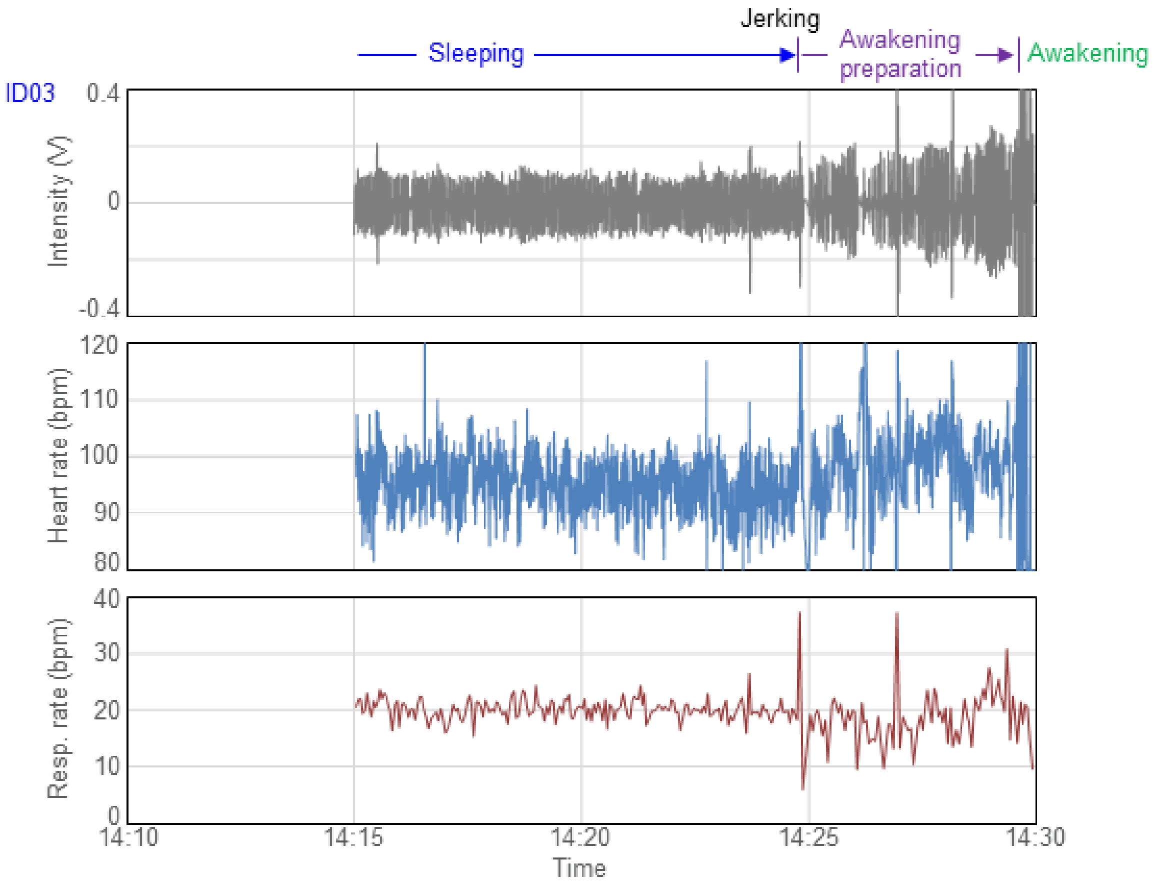

Jerking
Sleeping
Awakening preparation
Awakening
ID03
0.4
0
-0.4
Intensity (V)
120
110
100
90
80
Heart rate (bpm)
40
30
20
10
0
Resp. rate (bpm)
14:10
14:15
14:20
14:25
14:30
Time

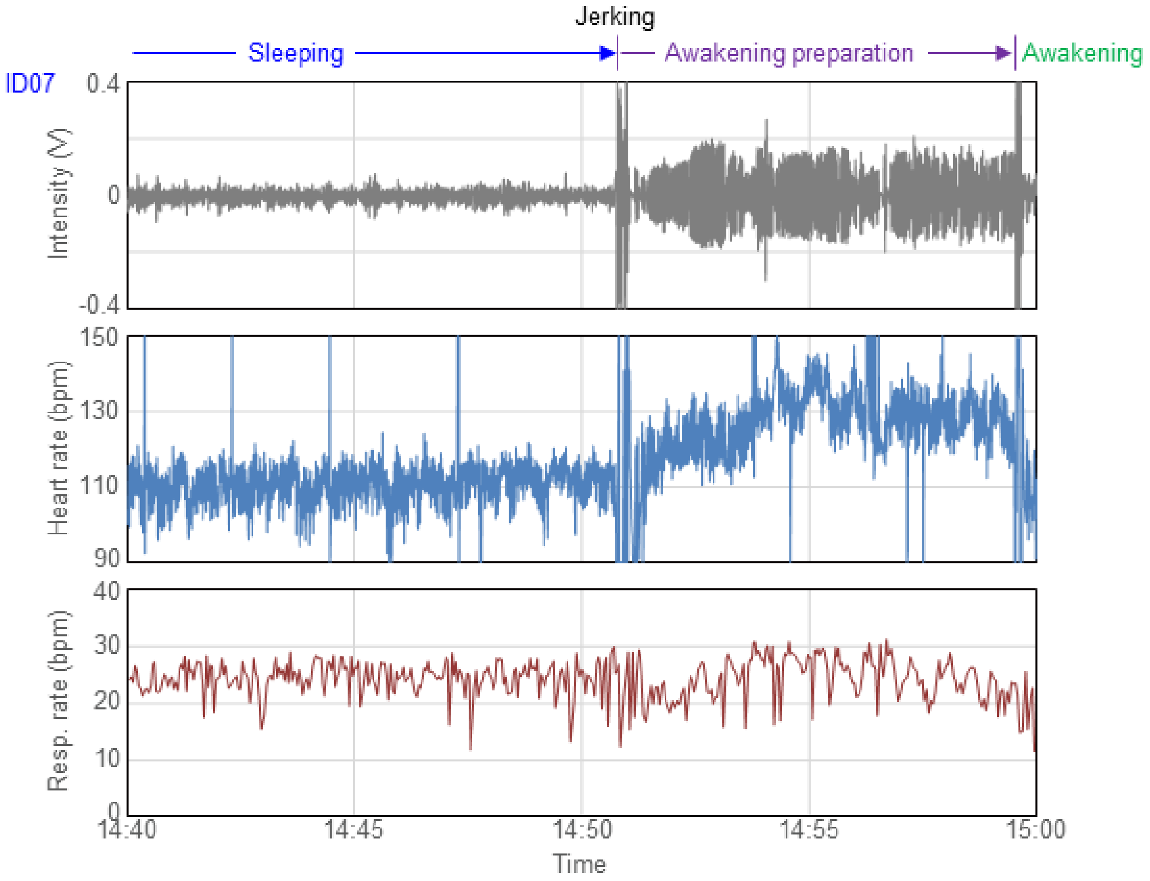

Jerking
Sleeping
Awakening preparation
Awakening
ID07
0.4
0
-0.4
Intensity (V)
150
130
110
90
Heart rate (bpm)
40
30
20
10
0
Resp. rate (bpm)
14:40
14:45
14:50
14:55
15:00
Time

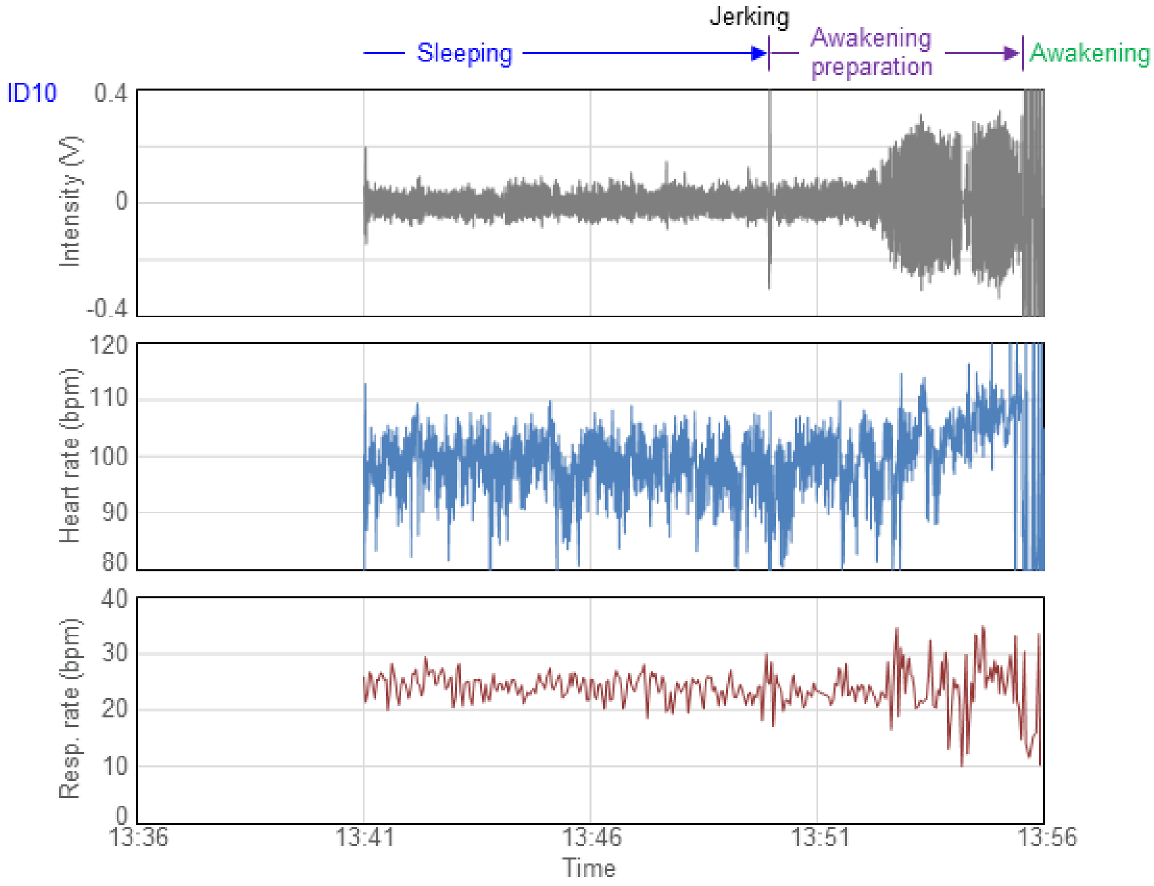
Jerking
Sleeping
Awakening preparation
Awakening
ID10
Intensity (V)
0.4
0
-0.4
Heart rate (bpm)
120
110
100
90
80
Resp. rate (bpm)
40
30
20
10
0
13:36
13:41
13:46
13:51
13:56
Time

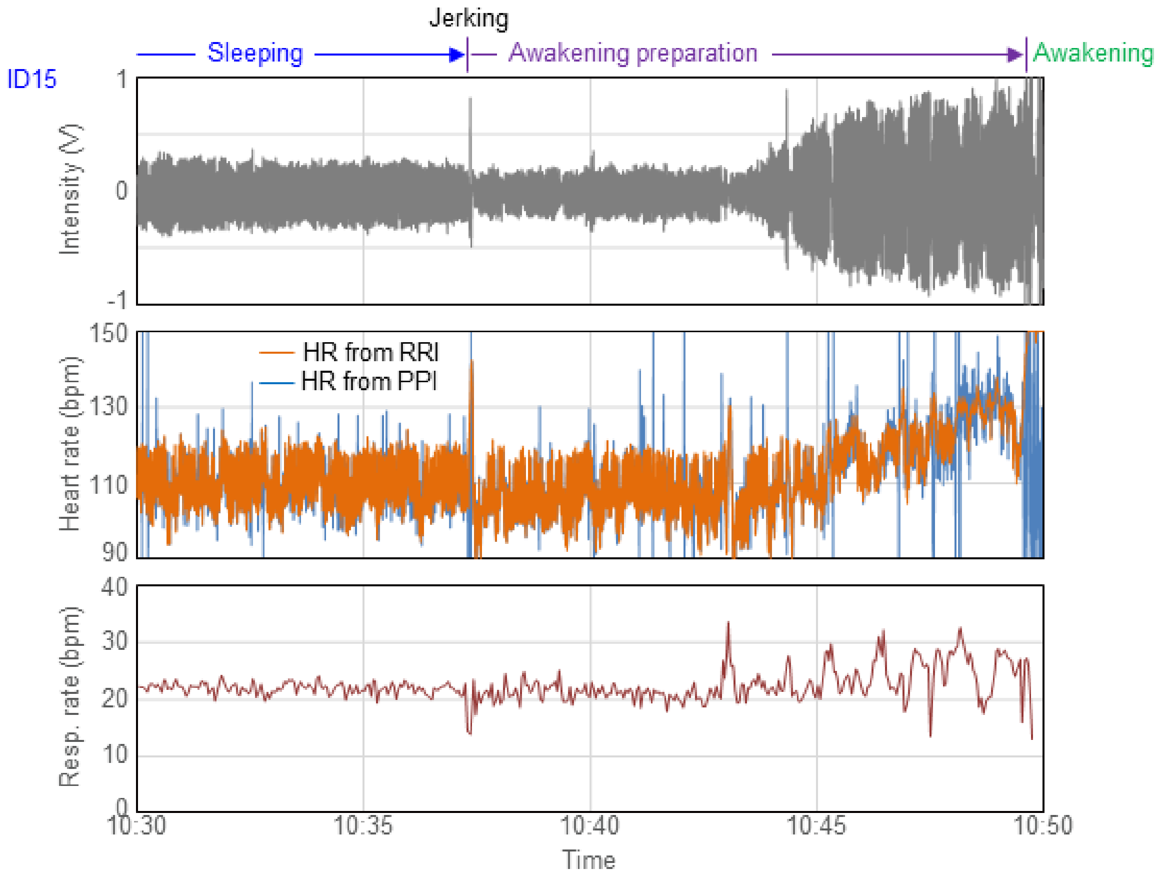
Jerking
Sleeping
Awakening preparation
Awakening
ID15
Intensity (V)
1
0
-1
HR from RRI
HR from PPI
Heart rate (bpm)
150
130
110
90
Resp. rate (bpm)
40
30
20
10
0
10:30
10:35
10:40
10:45
10:50
Time

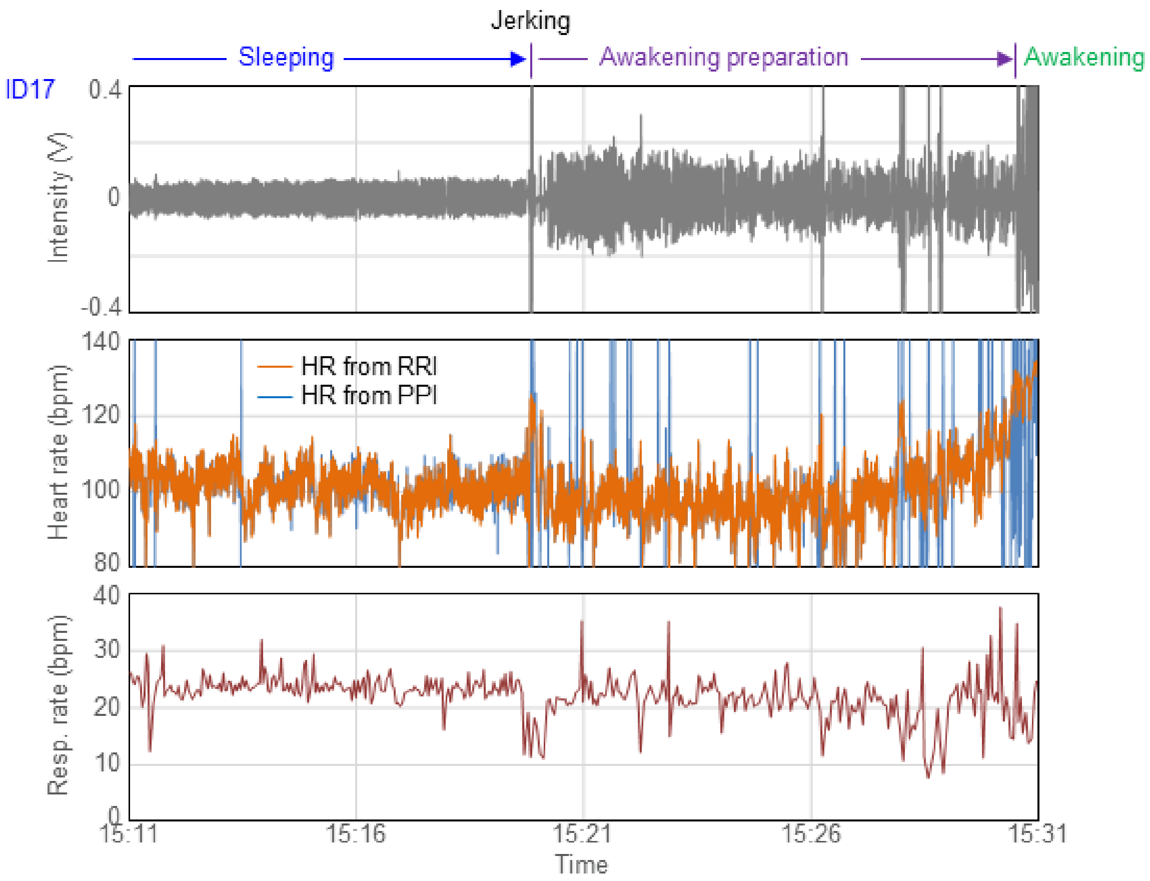

Supplementary Figure S2. Time dependence of respiration waveform, heart rate, and respiratory rate during awakening preparation stage for ID03, ID07, ID10, ID15, and ID17.
